# Azole Resistance and *ERG11* Mutation in Clinical Isolates of *Candida tropicalis*

**DOI:** 10.3390/jof11010024

**Published:** 2025-01-01

**Authors:** Adriele Celine Siqueira, Gisele Aparecida Bernardi, Lavinia Nery Villa Stangler Arend, Gabrielle Tomé Cordeiro, Daiane Rosolen, Fernanda Costa Brandão Berti, Amanda Maria Martins Ferreira, Thaís Muniz Vasconcelos, Bianca Cruz Neves, Luiza Souza Rodrigues, Libera Maria Dalla-Costa

**Affiliations:** 1 Instituto de Pesquisa Pelé Pequeno Príncipe (IPPPP), Curitiba 80250-060, Brazil; adriele.siqueira@outlook.com (A.C.S.); rosolen.daiane@gmail.com (D.R.); nandabrandao@hotmail.com (F.C.B.B.); thaismuniz6@hotmail.com (T.M.V.); 2 Faculdades Pequeno Príncipe (FPP), Curitiba 80230-020, Brazil; amandamariamferreira@gmail.com; 3 Laboratório Central do Estado do Paraná (LACEN-PR), São José dos Pinhais 83060-500, Brazil; gibernardi1509@gmail.com (G.A.B.); laviarend@gmail.com (L.N.V.S.A.); 4 Instituto de Química, Universidade Federal do Rio de Janeiro (UFRJ), Rio de Janeiro 21941-909, Brazil; gabicordeiro1994@gmail.com (G.T.C.); bcneves@iq.ufrj.br (B.C.N.)

**Keywords:** *Candida tropicalis*, Candidemia, Candiduria, azole resistance, *ERG11* gene, efflux pumps

## Abstract

We investigated the molecular mechanisms underlying azole resistance in seven *Candida tropicalis* isolates that caused candidemia and candiduria in Paraná, Brazil (2016–2022). Biofilm production, antifungal susceptibility testing, multilocus sequence typing, amplification and sequencing of *ERG11*, and quantification of *ERG11*, *MDR1*, and *CDR1* expression levels were performed. Notably, five isolates (71.4%) were from urine samples and two (28.6%) were from blood samples. All strains were biofilm producers, with levels ranging from moderate to strong. The minimum inhibitory concentration (MIC) values ranged from 8–>64 mg/L for fluconazole and 0.25–1 mg/L for voriconazole. All isolates had mutations in *ERG11*; Y132F and Y257N were predominant (71.4%), followed by Y132F and S154F (14.3%) and Y257H (14.3%). No differences in *ERG11* expression were found between the susceptible and resistant groups, but *MDR1* and *CDR1* were more highly expressed in the susceptible isolates. All the isolates contained previously unassigned diploid sequence types. The emergence of *C. tropicalis* azole resistance has been previously described in Brazil; however, the presence of resistant isolates in urine highlights the need for surveillance resistant strains in both urinary and invasive contexts. In our study mutations in *ERG11* were the main resistance mechanism identified in *C. tropicalis*.

## 1. Introduction

*Candida* species are ubiquitous fungi. They are commensal members of the endogenous microbiota of healthy individuals but are opportunistic pathogens that exploit susceptible hosts to establish their infections, primarily affecting immunocompromised individuals [1,2,3]. They comprise the majority of invasive fungal diseases (IFDs) in hospitalized patients, leading to high morbidity and mortality rates that can vary depending on the species responsible for the infection [4].

*Candida tropicalis* is considered one of the most virulent *Candida* species and is an important cause of nosocomial infections, more common than *Candida albicans* and other non-albicans species in developing countries such as India and some regions of Latin America, including Brazil [5,6,7]. It is on the list of priority groups of pathogens for research and development of new treatments by the World Health Organization, as it is associated with mortality rates of 55–60% in adults and 26–40% in children with invasive infections [8].

The increased incidence of antibiotic resistance in *C. tropicalis* contributes to its pathogenicity, making it challenging to treat [6]. Antifungal resistance represents a serious public health problem, given the limited number of antifungal agents available for the treatment of systemic infections and the adverse effects and toxicities that limit treatment in terms of increased dosage and prolonged use of drugs [9]. Owing to their low cost and low risk of adverse effects, azole drugs are one of the most widely used classes in treating *Candida* spp. infections, especially fluconazole [9,10]. However, because of the numerous resistance mechanisms developed by these microorganisms, the emergence of resistance to azole drugs has been reported in *C. tropicalis* isolates. Furthermore, the increasing rates of resistant *C. tropicalis* in urinary tract infections are a concern, as the presence of *Candida* spp. in urine is often associated with contamination or colonization of the host and is frequently overlooked [11,12].

Among the resistance mechanisms, structural alterations in the enzyme lanosterol 14α-demethylase (target of azoles), encoded by the *ERG11* gene, represent the most reported form of resistance in *Candida,* as they reduce the affinity of the drug for the target, thereby limiting its action. Resistance to azole drugs in *C. tropicalis* may also be related to upregulation of the *ERG11* gene due to mutations in regulatory genes, such as *UPC2,* and an overexpression of efflux pumps encoded by the *Candida* drug resistance 1 (*CDR1*) and multidrug resistance 1 (*MDR1*) genes [11,13,14].

In this study, we investigated the molecular mechanisms of azole resistance of *C. tropicalis* by characterizing mutations in the *ERG11* gene, as well as quantifying the expression levels of *ERG11*, *CDR1*, and *MDR1*. To the best of our knowledge, this is the first study to explore the molecular mechanisms underlying azole resistance in *C. tropicalis* isolates obtained from urine and blood samples in Brazil. Our findings highlight such resistance mechanisms, which are of significant epidemiological relevance and are useful for establishing stewardship programs and patient management.

## 2. Materials and Methods

### 2.1. Isolates and Clinical Setting

This retrospective study included clinical isolates stored in skimmed milk and frozen at −80 °C at the Central Laboratory of the State of Paraná, LACEN-PR (Reference Lab for State of Paraná) and Pelé Pequeno Príncipe Research Institute from August 2016 to August 2022. A total of 114 isolates of *C. tropicalis* were evaluated for susceptibility to azoles and seven of them showed resistance to fluconazole. A unique isolate from each patient was considered for this study. As a control group, clinical azole-susceptible isolates were used for comparison in the assessment of *ERG*11, *MDR*1, and *CDR*1 gene expression levels.

All microorganisms were identified through matrix-assisted laser desorption ionization time-of-flight mass spectrometry (MALDI-TOF MS) using a Microflex TM LT instrument (Bruker Daltonics, Billerica, MA, USA) [15].

### 2.2. Data Collection and Ethical Approval

This study was approved by the Institutional Review Board (IRB) of the participating center (IRB #6.481.883). We ensured the anonymity of the patients involved.

### 2.3. Biofilm Formation

The biofilm production capacity of the isolates was measured using 96-well flat-bottomed microtiter plates and crystal violet dye (1%, *v*/*v*), as previously described by our group [16].

### 2.4. Antifungal Susceptibility Testing

Antifungal susceptibility testing of all *C. tropicalis* isolates was carried out using the broth microdilution technique for micafungin (0.0078–8.0 µg/mL), fluconazole (0.125–64 µg/mL), voriconazole (0.016–1.0 µg/mL), and amphotericin B (0.063–8.0 µg/mL), according to the European Committee for Antimicrobial Susceptibility Testing (EUCAST) [17]. To ensure the test quality, two reference strains, *Candida parapsilosis* ATCC 22019 and *Candida krusei* ATCC 6258, were included as controls for each antifungal susceptibility test. The minimum inhibitory concentration (MIC) results were interpreted according to the EUCAST breakpoints [17].

### 2.5. Genomic DNA Extraction, Polymerase Chain Reaction, and ERG11 Sequencing

Genomic DNA was extracted using a previously described in-house technique, using guanidium thiocyanate and a zymolase buffer to digest the fungal cell wall and initiate cell lysis [18]. Then, polymerase chain reaction (PCR) was performed to amplify *ERG11* in all azole-resistant *C. tropicalis* isolates, using two pairs of primers: forward 1—5′-TCA CAG TTA TAG ACC CAC AAG G-3′ and reverse 1—5′-TCA CCG CTT TCT CTT CTC T-3′ and forward 2—5′-AAG GTT TCA CCC CAA TCA ACT T-3′ and reverse 2—5′-AAC ATT AGA AGT TCT TGC TAA G-3′. The concentrations of reagents and amplification conditions were as described previously [19]. The amplification products were analyzed via agarose gel electrophoresis (1.5% *w*/*v*) and purified using the ExoSAP-IT PCR Product Cleanup Reagent (Thermo Fisher Scientific, Waltham, MA, USA). Sequencing was performed on an ABI 3500 automated sequencer (Applied Biosystems, Waltham, MA, USA) using the same primers used for PCR. Finally, the *ERG11* sequences were manually aligned using Bioedit v. 7.2 software and compared with the *C. tropicalis* MYA3404 (GenBank accession number XM_002550136) reference sequence to assess the presence of mutations.

### 2.6. Three-Dimensional Structural Modeling of C. tropicalis Lanosterol 14α-Demethylase Mutant Enzymes

The SWISS-MODEL platform was used to model the three-dimensional structure of all Erg11 protein mutants identified in this study. The template was the lanosterol 14α-demethylase enzyme from *C. albicans*, whose sequence is available in UniProt (accession number: A0A0A0Y3Q3). This enzyme was chosen based on its structural definition using AlphaFold, which provided a reliable model [20]. Using ClustalW, the mutant protein sequences were aligned with the protein model [21]. This ensured that the molecular geometry and structural conservation of the critical regions were considered. PyMOL 2.3 was used to visualize the structures after modeling and Ramachandran plots for each mutant were used to assess the model quality [22].

### 2.7. RNA Isolation and Quantitative Reverse Transcription PCR

The total RNA was extracted using TRIzol™ (guanidine thiocyanate and phenol). First, 10 µL of microorganisms was transferred into 1 mL of PBS buffer using a loaded bacteriological loop. The contents were centrifuged at 5000× *g* for 10 min, and the supernatant was removed. The pellet was used for enzymatic treatment with 100 µL of zymolyase buffer and 10 µL of lyticase, incubated for 30 min at 30 °C, centrifuged at 5000× *g* for 10 min, and the supernatant was discarded. The pellet was resuspended in 1 mL of TRIzol™, homogenized repeatedly, and centrifuged at 12,000× *g* for 10 min at 4 °C; the entire supernatant was transferred to a new microtube and left to stand for 5 min at 20–25 °C (room temperature). To separate the phases, 200 µL of chloroform was added, shaken vigorously for 15 s, and allowed to stand for 5 min at room temperature. The contents were centrifuged at 12,000× *g* for 15 min at 4 °C and the entire upper aqueous phase containing the RNA was transferred to a new microtube. Then, 500 µL of ice-cold isopropanol was added, homogenized by inversion, and incubated overnight at −80 °C. To precipitate the RNA, we centrifuged at maximum speed for 20 min at 4 °C and discarded the supernatant. The pellet was washed with 1 mL of ice-cold 75% ethanol, shaken until it detached from the tube wall, and centrifuged at 7500× *g* for 5 min at 4 °C. Without touching the walls of the tube, the ethanol was removed with a pipette, and 1 mL of ice-cold absolute ethanol was added, again shaken and centrifuged at 7500× *g* for 5 min at 4 °C. All the ethanol was removed, and the pellet was allowed to dry for approximately 30 min and resuspended in 20–60 µL of ultrapure water. A reverse transcription of the extracted RNA samples was performed using the Transcriptor Universal cDNA Master Mix (Sigma Aldrich, St. Louis, MO, USA) according to the manufacturer’s instructions. The expression levels of *ERG11*, *MDR1*, and *CDR1* were assessed through a quantitative reverse transcription PCR (RT-qPCR) using ABI 7500 (Applied Biosystems) and PowerUP SYBR Green Master Mix (Thermo Fisher Scientific), according to previously described reaction parameters [23], in triplicate using the *ACT1* gene as a normalizer. The transcription levels of the azole-resistant isolates were compared with the expression levels of seven sensitive isolates (control group). Primers used for RT-qPCR have been described previously [13].

### 2.8. Multilocus Sequence Typing

Multilocus sequence typing (MLST) was performed using six housekeeping genes for *C. tropicalis* isolates, determined according to the PubMLST website (http://pubmlst.org/organisms/candida-tropicalis, accessed on 20 May 2024). The primers and PCR assays used were described previously [24,25]. After amplification, the PCR products were analyzed via agarose gel electrophoresis (1.5% *v*/*w*) and purified using ExoSAP-IT PCR Product Cleanup Reagent (Thermo Fisher Scientific). The same PCR primers were used for sequencing on an ABI 3500 automated sequencer (Applied Biosystems), and the electropherograms were analyzed and aligned using Bioedit v. 7.2 software. Allele were assigned and diploid sequence types (DSTs) determined using the *C. tropicalis* MLST database (http://pubmlst.org/ctropicalis/, accessed on 14 July 2024).

## 3. Results

### 3.1. Clinical Information

In total, 114 isolates of *C. tropicalis* were screened during the study period: 56 were isolated from blood samples, 34 from urine samples, eight from respiratory samples and 16 from other clinical samples. Of these, seven clinical isolates of azole-resistant *C. tropicalis* were included in this study. Most clinical isolates (71.4%, n = 5) were recovered from the urine samples, whereas 28.6% (n = 2) were from the blood samples. The age of the patients with resistant isolates ranged from 5 to 69 years old (mean 41.6 ± SD 25.4), of whom five were men and two were women (ratio 5:2) (Table 1).

### 3.2. Biofilm Production

All isolates in this study demonstrated biofilm-forming capacity. Six (85.7%) were classified as moderate producers and one (14.3%) as a strong producer (Table 1).

### 3.3. Antifungal Susceptibility

One isolate (14.3%) was highly resistant to fluconazole (MIC > 64 mg/L), whereas six isolates (85.7%) showed low resistance (MIC 8–64 mg/L). For voriconazole, MIC values ranged from 0.25 to 1 mg/L. All isolates were susceptible to micafungin and amphotericin B (Table 1).

### 3.4. Missense Mutations in the ERG11 Gene

Compared to the wild-type isolate of *C. tropicalis* (MYA-3404), all azole-resistant isolates included in this study had mutations in the *ERG11* gene, including silent mutations that did not alter the conformation of the encoded enzymes C75C (C225T), L88L (A264G), I261I (T783C), K454K (A1362G), and I518I (C1554T). In addition, four homozygous missense mutations linked to resistance were detected, of which the Y132F (A395T) and Y257N (T769A) mutations were the most common—present in 71.4% (n = 5) of the isolates. The Y132F (A395T) and S154F (C461T) mutations were found in one isolate (14.3%), and one isolate harbored only the Y257H (T769C) mutation. Notably, no novel missense mutations were detected. In the sensitive isolates, included as a control group for gene expression, only silent mutations were observed (Table 1).

### 3.5. Three-Dimensional Structural Modeling of C. tropicalis Lanosterol 14α-Demethylase Mutant Enzymes

**Figure 1:**
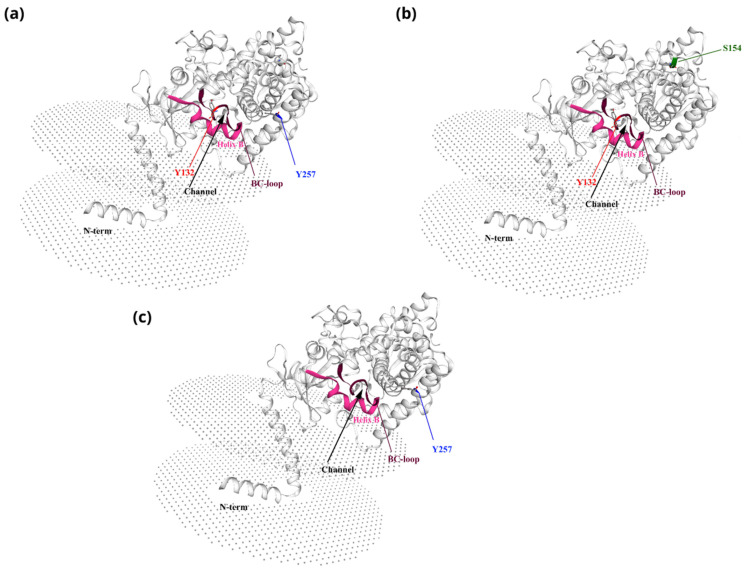
Three-dimensional models of *C. tropicalis* Erg11 mutant enzymes. The modeling of the three-dimensional structure of the Erg11 mutant proteins Ctr1 (Y132F/Y257N) (**a**), Ctr4 (Y132F/S154F) (**b**) and Ctr7 (Y257H) (**c**) was carried out by SWISS-MODEL, using the Erg11 enzyme from *C. albicans* (Uniprot–A0A0A0Y3Q3), elucidated via AlphaFold. Helix B is shown in pink, followed by the BC loop in brown, and the substrate access channel in black. Substituted residues are shown in red (Y132), in blue (Y257), and in green (S154).

We constructed three-dimensional structural models of *C. tropicalis* lanosterol 14α-demethylase mutant enzymes identified in this study. The results showed that the models were reliable, as more than 97% of the amino acid residues were within the permitted areas. The accuracy of the generated models was confirmed by root mean square deviation values of 0.139 Å, 0.096 Å, and 0.141 Å for the mutants Ctr1 (Y132F/Y257N), Ctr4 (Y132F/S154F), and Ctr7 (Y257H), respectively (Figure 1). Although there were no remarkable differences between the general structure of the amino acid sequence of wild-type and mutant lanosterol 14α-demethylase, all the substitutions identified in this study are located in or near the azole-binding site of the enzyme and can affect the affinity [7,26,27,28].

### 3.6. Expression Levels of ERG11, MDR1, and CDR1

**Figure 2:**
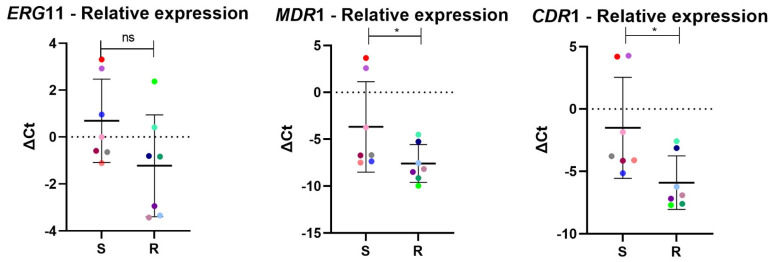
Expression levels of *ERG11* and efflux pump genes. Expression levels of *ERG11*, *MDR1*, and *CDR1* genes in azole-resistant (R) and azole-susceptible (S) groups of *C. tropicalis*. * *p* < 0.05; ns, non-significant. The isolates are represented by colored dots. Dots with the same color in the graphs indicate the same isolate.

RT-qPCR analysis revealed no differences in the expression levels of the *ERG11* gene between azole-resistant and azole-susceptible isolates. Conversely, genes encoding efflux pumps (*MDR1* and *CDR1*) were significantly more highly expressed in susceptible isolates than in resistant isolates (Figure 2).

### 3.7. Multilocus Sequence Typing Analysis

Analysis of the DNA fragments from the six target genes used in MLST of *C. tropicalis* identified five novel alleles: two in the *ICL1* gene and one each in *SAPT4*, *XYR1*, and *ZWF1a* genes. All identified DSTs were novel to the *C. tropicalis* MLST database, and four different allelic profiles were assigned among the samples, totaling five clones, as shown in Table 2.

## 4. Discussion

*C. tropicalis* is an opportunistic pathogen that causes invasive infections with high mortality, especially in immunocompromised individuals [13,14]. The incidence may vary according to geographical region and other factors, but the frequency of infections caused by this microorganism has increased over the years. Currently, the species is among the most isolated yeasts in some regions of Asia and Latin America [11,29,30]. In Brazil, studies on samples from candiduria and candidemia in adult and pediatric patients have shown that *C. tropicalis* is among the three most prevalent species [2,31,32].

In this study, all isolates produced biofilms ranging from moderate (85.7%) to strong (14.3%). Compared with other species, *C. tropicalis* has a greater capacity for adhesion and biofilm biomass [33,34]. This efficiency in biofilm formation, along with other virulence mechanisms, contributes to *C. tropicalis* being one of the most pathogenic species in the *Candida* genus, as it allows fungal cells to be less susceptible to the host’s immune responses and antifungal treatment [7,34,35].

In addition to virulence, another factor of concern in *C. tropicalis* infections is the increasing rate of resistance to azole drugs, which are one of the most widely used antifungal classes for the treatment of clinical infections by *Candida* spp., owing to low cytotoxicity and costs related to treatment [29,36,37,38].

Most studies on resistance in *C. tropicalis* have been conducted using strains isolated from candidemia, which is considered a major form of invasive infection. However, a previous study that found a high resistance rate to azoles in isolates from candiduria suggested that his resistance is observed earlier in this type of specimen than in strains from invasive infections [12]. The presence of *Candida* spp. in urine is often associated with host contamination or colonization. However, cases of candiduria may be associated with invasive infections and an increased risk of death, and considering that many surveillance studies exclude candiduria isolates, antifungal resistance in these samples may be underestimated [12,29,39]. In this study, 71.4% of fluconazole-resistant isolates were from urinary tract infections and 28.6% were from bloodstream infections. One of the limitations of our study is that these urinary isolates could not be unambiguously attributed to the infection or colonization sources. The age of the patients affected by candiduria ranged from 5 to 69 years, with only two patients older than 60 years, and the majority were male (n = 3/5) (Table 1). These observations are noteworthy, as previous studies have shown that people over 60 years of age and women are most likely to develop candiduria [12,31].

Azole resistance in *C. tropicalis* has been observed in approximately 13–40% of isolates in China [14,40], approximately 6% in the United States [41], and 6–20% in European countries [42,43]. In Brazil, a study of 200 bloodstream isolates of *C. tropicalis* from six states examined temporal trends in azole resistance and showed a decrease in azole susceptibility over the years, with non-susceptibility rates of 4% for fluconazole and 3.5% for voriconazole [44].

In addition, we evaluated the potential molecular mechanisms involved in azole resistance (to fluconazole and voriconazole) in seven clinical isolates of *C. tropicalis* collected between 2016 and 2022. In agreement with other studies, we observed that resistance to azole drugs in these isolates seemed to be mainly related to mutations in the *ERG11* gene, which alters the conformation of the lanosterol 14-alpha-demethylase enzyme, ultimately decreasing its affinity for the drug [45].

A total of 31 mutations in the *ERG11* gene have previously been described to be associated with resistance in *C. tropicalis* isolates [11,12]. In this study, the Y132F (A395T) and Y257N (T769A) mutations were the most prevalent, being found in 71.4% (n = 5) of the isolates resistant to fluconazole (MIC 16–64 mg/L) and voriconazole (0.25–0.5 mg/L) (Figures S1 and S2). Only one isolate (14.3%) carried the Y132F (A395T) and S154F (C461T) mutations (MIC > 64 mg/L for fluconazole and 1 mg/L for voriconazole) (Figure S3), differing from other studies, which reported them as being the most frequent mutations in *C. tropicalis.* [13,40,45]. A previous mutagenesis study analyzing the Y132F (A395T) and S154F (C461T) substitutions individually concluded that only the Y132F (A395T) mutation is responsible for azole resistance [40]. Consistent with this, a later study using a three-dimensional model of the Erg11 enzyme from *C. tropicalis* reported that the Y132F mutation, located within the active site of the enzyme, significantly affects the azole resistance profile. In contrast, the S154F substitution, located on the surface of the enzyme near the heme group, is unlikely to be directly associated with resistance [27]. However, despite being located on the surface of the protein, the S154C mutation, in combination with the Y132F mutation, has been shown to decrease the binding energy to azoles, thereby decreasing the affinity of the enzyme for the ligand [28]. Finally, one isolate (14.3%) harbored only the Y257H (T769C) mutation, which is located in helix G actually outside the active site itself, and which has previously been associated with reduced susceptibility, including in isolates of environmental origin (Figure S2) [7]. In the control group isolates (sensitive), only silent mutations were found, which do not alter the enzyme’s conformation or interfere with the resistance profile (Table 1).

Other important mechanisms previously described to be responsible for azole resistance in *C. tropicalis* include the positive regulation of the *ERG11* gene, which is a consequence of mutations in regulatory genes, and the overexpression of genes encoding important efflux pumps. Two types of efflux pumps cause azole resistance in these isolates: transporters of the ATP-binding cassette (ABC) family of proteins encoded by the *CDR1* gene and transporters of the major facilitator superfamily (MFS) encoded by the *MDR1* gene. Overexpression of these genes leads to an increase in the number of efflux pumps in the membrane, thereby reducing drug accumulation in the intracellular environment [14,28,36].

In our study, no differences in the expression of *ERG11* were observed between the susceptible and azole-resistant groups. In contrast, the *CDR1* and *MDR1* genes, which encode efflux pumps, showed decreased expression in the resistant isolates compared to the susceptible ones. All resistant isolates had missense mutations in the *ERG11* gene. A study of *C. tropicalis* in China found no significant differences in the expression of *CDR1* and *MDR1* between susceptible and resistant isolates. However, the expression of these genes was significantly higher in resistant isolates with wild-type *ERG11* than in those with missense mutations in *ERG11* [40]. This differential expression pattern has also been observed in *C. albicans* isolates [46]. A possible explanation is that in isolates with a mutated drug target that makes antifungal activity ineffective, the cell may conserve energy by reducing the expression of efflux pumps. However, although we observed significantly higher expression levels of *MDR1* and *CDR1* in the sensitive group, gene expression varied between isolates. Due to the limited number of samples, statistical significance may be directly influenced. Therefore, for these data to be representative and to further explore the mechanisms of resistance to azoles, studies with a larger number of isolates will be necessary.

In this study, MLST was used for molecular typing of the isolates. Because it allows the comparison of data from the same study, as well as from different geographical regions, this technique has become an essential tool for epidemiological studies and research into genetic diversity [47]. Four distinct allelic profiles were identified among the clinical isolates of *C. tropicalis*, two of which covered more than one isolate: one profile with two isolates and the other with three isolates considered to be clones. Although the allelic profiles differed, MLST analysis revealed that this difference was only due to the *MDR1* allele, demonstrating their proximity (Table 2). In addition, the five isolates belonging to these two allelic profiles contained the same mutations in the *ERG11* gene (Y132F and Y257N), differentiating them from the other isolates (Table 1).

## 5. Conclusions

The resistance to azoles in *C. tropicalis* has raised increasing public concern worldwide, including in the context of health, highlighting the connection between human, animal, and environmental health. Urine samples are generally excluded from surveillance studies; however, the urinary tract may be an important reservoir for the acquisition, emergence, and spread of resistance. Our study revealed that the Y132F and Y257N mutations in *ERG11* are important mechanisms conferring resistance to azoles in *C. tropicalis*. Understanding these mechanisms can contribute to better management of infections, treatment of patients, and prevention of resistance dissemination.

## Figures and Tables

**Table 1:** Clinical, phenotypic, and molecular characteristics of seven azole-resistant *C. tropicalis* isolates and the control group.

Isolate ID	Sample	Date of Isolation	Institution	City	Biofilm Production	Mutation in the *ERG*11 Gene	MLST	Antifungal Susceptibility Testing (mg/L)			
								FLU	VOR	MIC	AMB
**Resistant isolates**											
Ctr1	Blood	26 March 2022	A	Curitiba	POS++	C75C, L88L, **Y132F, Y257N**	new DST	16	0.5	0.015	≤0.06
Ctr2	Urine	12 March 2022	B	Londrina	POS++	C75C, L88L, **Y132F, Y257N**	new DST	16	0.5	0.007	≤0.06
Ctr3	Urine	15 March 2022	B	Londrina	POS++	C75C, L88L, **Y132F, Y257N**	new DST	64	0.5	0.015	≤0.06
Ctr4	Urine	15 March 2022	B	Londrina	POS++	**Y132F, S154F**	new DST	>64	1	0.015	≤0.06
Ctr5	Urine	11 February 2021	A	Curitiba	POS++	C75C, L88L, **Y132F, Y257N**	new DST	16	0.25	0.015	≤0.06
Ctr6	Urine	5 May 2017	C	Curitiba	POS++	C75C, L88L, **Y132F, Y257N**	new DST	16	0.5	0.015	≤0.06
Ctr7	Blood	24 April 2022	C	Curitiba	POS+++	C75C, L88L, I261I, K453K, I518I, **Y257H**	new DST	8	0.25	0.015	≤0.06
**Sensitive isolates (control group)**											
Ctr8	Blood	30 September 2016	C	Curitiba	POS+++	C75C, L88L, H183H, K454K, I518I,	new DST	0.5	≤0.016	0.015	≤0.06
Ctr9	Blood	11 October 2017	C	Curitiba	POS+++	C75C, L88L, K454K, I518I	new DST	0.25	≤0.016	0.015	≤0.06
Ctr10	Blood	29 June 2018	C	Curitiba	POS+++	I261I	new DST	0.50	≤0.016	0.015	0.125
Ctr11	Blood	8 February 2019	C	Curitiba	POS+++	C75C, L88L, I261I	new DST	0.50	≤0.016	0.015	≤0.06
Ctr12	Blood	18 November 2019	C	Curitiba	POS+++	C75C, L88L	new DST	0.50	≤0.016	0.015	≤0.06
Ctr13	Blood	8 August 2020	C	Curitiba	POS+++	C75C, L88L, I261I, K454K, I518I	new DST	0.25	≤0.016	0.015	≤0.06
Ctr14	Blood	22 September 2021	C	Curitiba	POS+++	C75C, L88L, K454K, I518I	new DST	0.25	≤0.016	0.015	0.125

ID, identification; Ctr, *C. tropicalis*; POS, positive; DST, diploid sequence type; FLU, fluconazole; VOR, voriconazole; MIC, micafungin; AMB, amphotericin B. The missense mutations that alter the conformation of the enzyme are bolded. The letters A, B, and C refer to the healthcare institutions from which the isolates were obtained.

**Table 2:** *C. tropicalis* allelic profiles identified using the pubMLST database.

*C. tropicalis*	Housekeeping Genes						DSTs
	*ICL1*	*MDR1*	*SAPT2*	*SAPT4*	*XYR1*	*ZWF1a*	
**Resistant isolates**							
**Ctr1**	**1**	**152**	**1**	**11**	**2**	**3**	**new**
**Ctr2**	**1**	**27**	**1**	**11**	**2**	**3**	**new**
**Ctr3**	**1**	**152**	**1**	**11**	**2**	**3**	**new**
Ctr4	new	7	12	46	48	22	new
**Ctr5**	**1**	**27**	**1**	**11**	**2**	**3**	**new**
**Ctr6**	**1**	**27**	**1**	**11**	**2**	**3**	**new**
Ctr7	new	143	3	new	new	new	new
**Sensitive isolates (control group)**							
Ctr8	1	7	4	9	50	1	new
Ctr9	1	17	2	14	2	3	new
Ctr10	1	7	4	6	52	4	238
Ctr11	1	7	3	6	192	10	new
Ctr12	1	1	3	new	new	1	new
Ctr13	42	1	18	1	5	1	new
Ctr14	1	17	2	14	9	3	new

DSTs, diploid sequence types. Isolates with the same allelic profile (DSTs) are bolded.

## Data Availability

All data that support the conclusions of this study are available from the corresponding author upon a reasonable request.

## Supplementary Materials

The following supporting information can be downloaded at: https://www.mdpi.com/article/10.3390/jof11010024/s1, Figure S1: Multiple sequence alignment of the Y132F mutation in the *ERG*11 gene of sensitive and resistant *C. tropicalis* isolates; Figure S2: Multiple sequence alignment of the Y257N/H mutations in the *ERG*11 gene of sensitive and resistant *C. tropicalis* isolates; Figure S3: Multiple sequence alignment of the S154F mutation in the *ERG*11 gene of sensitive and resistant *C. tropicalis* isolates.
