# Supplementary figures and images for: Azole Resistance and ERG11 Mutation in Clinical Isolates of Candida tropicalis
Source: J Fungi (Basel). 2025 Jan 1;11(1):24. doi: 10.3390/jof11010024 (PMC11767116; doi:10.3390/jof11010024)

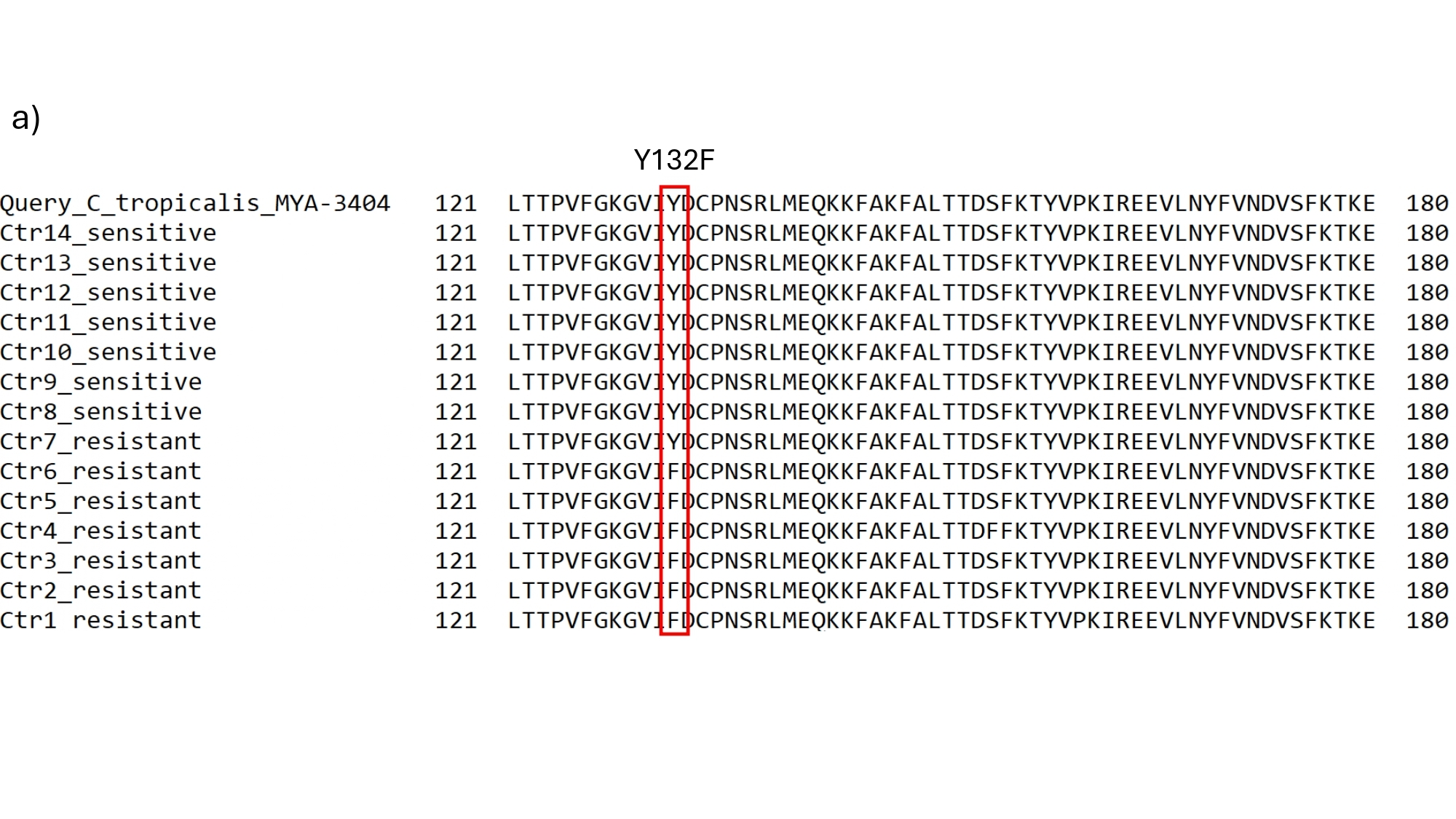

Supplement: Supplementary file 1 [file jof-11-00024-s001.zip › Figure S1.jpg]

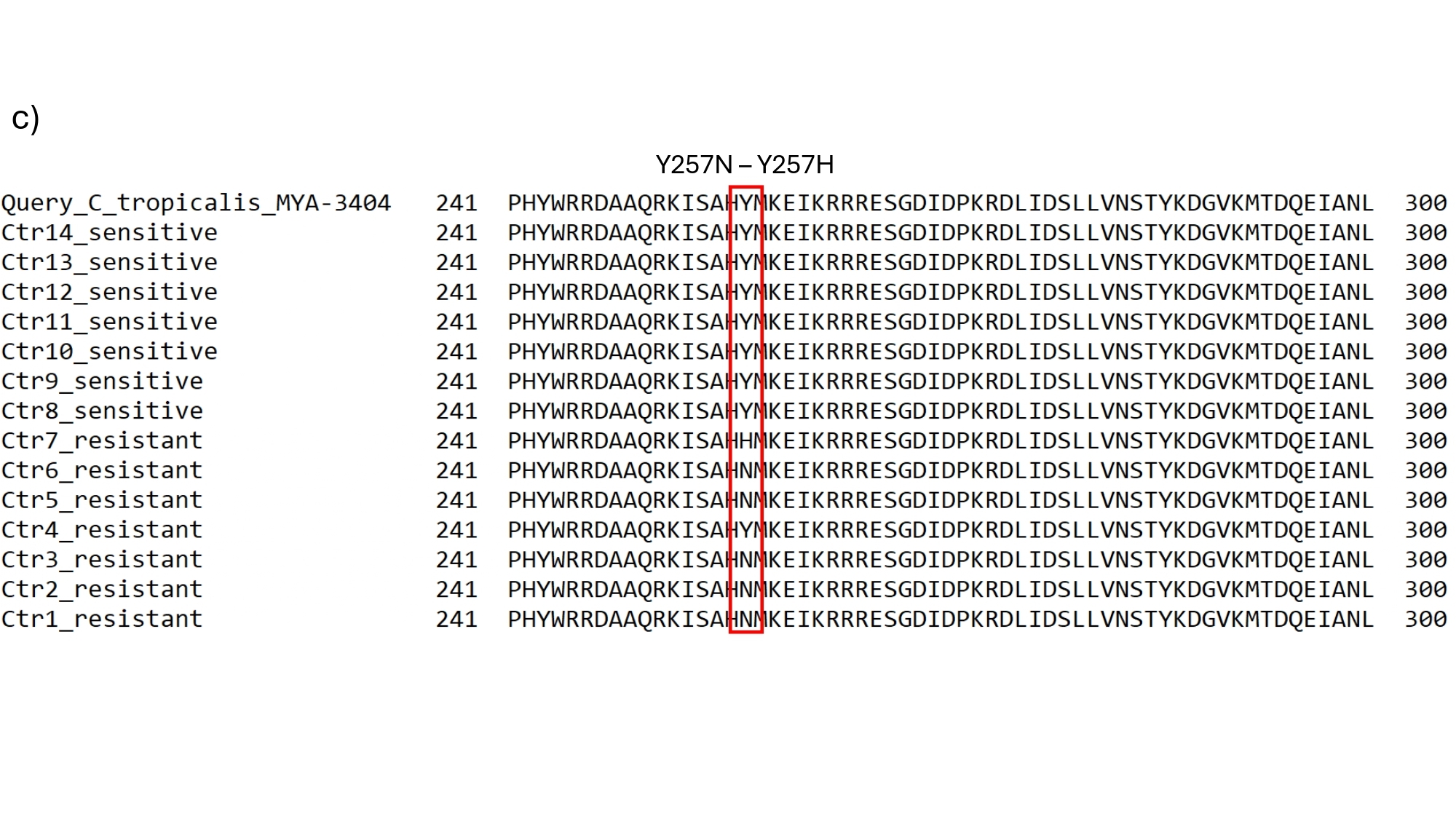

Supplement: Supplementary file 1 [file jof-11-00024-s001.zip › Figure S2.jpg]

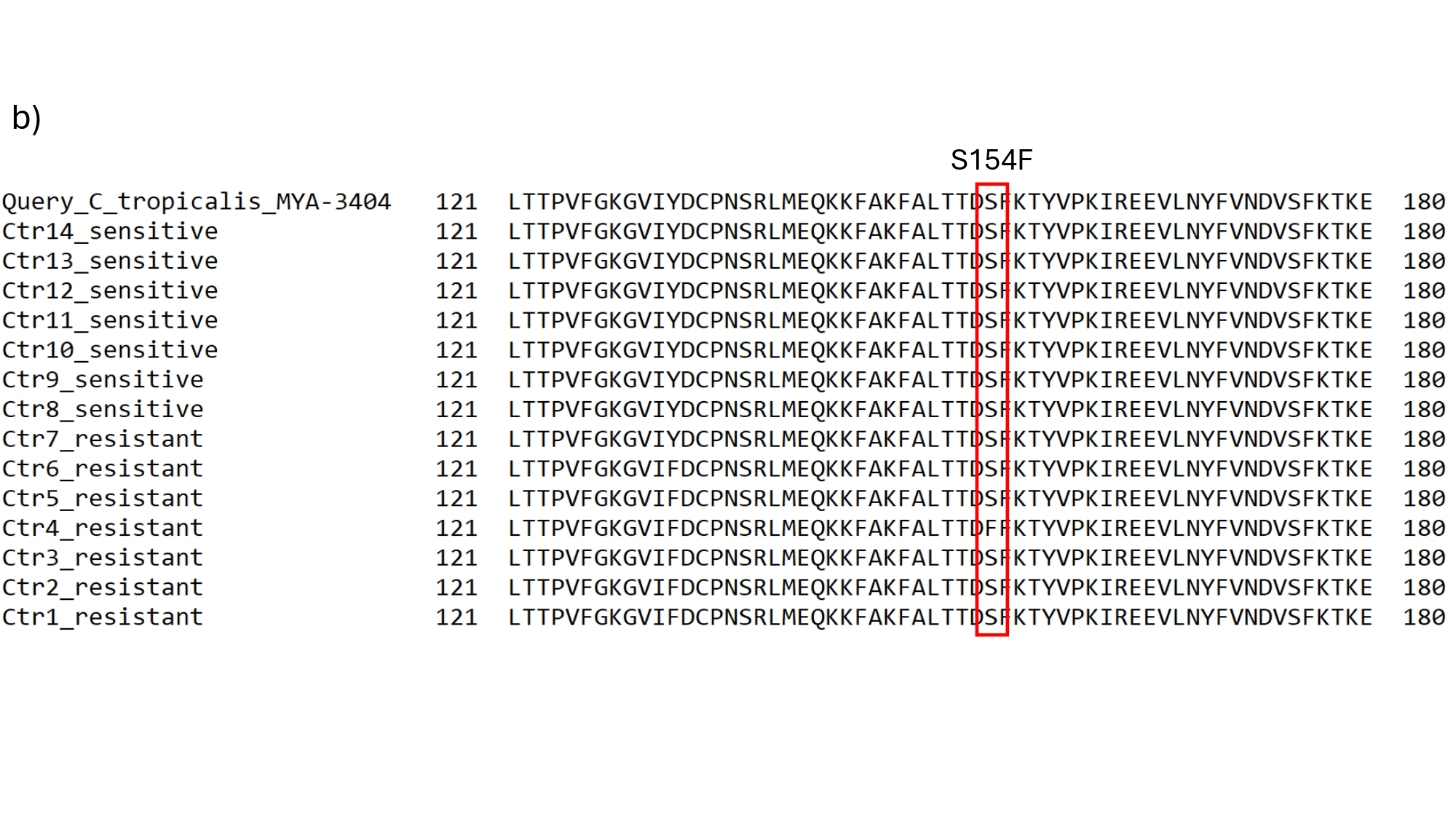

Supplement: Supplementary file 1 [file jof-11-00024-s001.zip › Figure S3.jpg]
